# Safety and Immunogenicity of M2-Deficient, Single Replication, Live Influenza Vaccine (M2SR) in Adults

**DOI:** 10.3390/vaccines9121388

**Published:** 2021-11-24

**Authors:** Joseph Eiden, Gilad Gordon, Carlos Fierro, Renee Herber, Roger Aitchison, Robert Belshe, Harry Greenberg, Daniel Hoft, Yasuko Hatta, Michael J. Moser, Magdalena Tary-Lehmann, Yoshihiro Kawaoka, Gabriele Neumann, Paul Radspinner, Pamuk Bilsel

**Affiliations:** 1 Celyn Consulting, LLC, Lewes, DE 19958, USA; jeiden@flugen.com; 2 ORRA Group, LLC, Boulder, CO 80305, USA; ggordon@orragroup.com; 3 Johnson County Clin-Trials, Lenexa, KS 66219, USA; cfierro@jcct.com; 4 FluGen, Inc., Madison, WI 53711, USA; rherber@flugen.com (R.H.); yhatta@flugen.com (Y.H.); mmoser@flugen.com (M.J.M.); pradspinner@flugen.com (P.R.); 5 North Rim Consulting, Longmont, CO 80504, USA; raitchison@northrimgroup.com; 6 Department of Internal Medicine, Saint Louis University, St. Louis, MO 63104, USA; belsherb@gmail.com (R.B.); hoftdf@slu.edu (D.H.); 7 Department of Molecular Microbiology, Saint Louis University, St. Louis, MO 63104, USA; 8 Department of Medicine, Division of Gastroenterology and Hepatology, Stanford University, Stanford, CA 94305, USA; hbgreen@stanford.edu; 9 Department of Microbiology and Immunology, Stanford University, Stanford, CA 94305, USA; 10 Cellular Technology Limited, Shaker Heights, OH 44122, USA; magda.tary-lehmann@immunospot.com; 11 Influenza Research Institute, University of Wisconsin, Madison, WI 53711, USA; yoshihiro.kawaoka@wisc.edu (Y.K.); gabriele.neumann@wisc.edu (G.N.)

**Keywords:** influenza, vaccine, clinical trial, M2SR, immunity

## Abstract

M2SR (M2-deficient single replication) is an investigational live intranasal vaccine that protects against multiple influenza A subtypes in influenza-naïve and previously infected ferrets. We conducted a phase 1, first-in-human, randomized, dose-escalation, placebo-controlled study of M2SR safety and immunogenicity. Adult subjects received a single intranasal administration with either placebo or one of three M2SR dose levels (10^6^, 10^7^ or 10^8^ tissue culture infectious dose (TCID_50_)) expressing hemagglutinin and neuraminidase from A/Brisbane/10/2007 (H3N2) (24 subjects per group). Subjects were evaluated for virus replication, local and systemic reactions, adverse events (AE), and immune responses post-vaccination. Infectious virus was not detected in nasal swabs from vaccinated subjects. At least one AE (most commonly mild nasal rhinorrhea/congestion) was reported among 29%, 58%, and 83% of M2SR subjects administered a low, medium or high dose, respectively, and among 46% of placebo subjects. No subject had fever or a severe reaction to the vaccine. Influenza-specific serum and mucosal antibody responses and B- and T-cell responses were significantly more frequent among vaccinated subjects vs. placebo recipients. The M2SR vaccine was safe and well tolerated and generated dose-dependent durable serum antibody responses against diverse H3N2 influenza strains. M2SR demonstrated a multi-faceted immune response in seronegative and seropositive subjects.

## 1. Introduction

Influenza viruses cause respiratory disease and additional medical complications, resulting in over 200,000 hospitalizations and 12,000 to 61,000 deaths per year in the United States [1]. Annual influenza vaccination is the primary means of preventing influenza and its complications. The CDC recommends seasonal influenza vaccination for all individuals aged 6 months and older. However, the vaccine effectiveness (VE) of current licensed vaccines is sub-optimal, ranging from 60% in years in which there is a good match between vaccine and circulating strains to as low as 6% in years in which there is a mismatch [2]. VE against the H3N2 subtype is especially problematic, accounting for higher hospitalization rates and excess mortality compared with H1N1 and influenza B infections [3]. Furthermore, concerns have been raised regarding waning of protection within the course of a single influenza season [4,5,6,7]. Thus, there is a need for new effective influenza vaccines that induce broader, cross-reactive and durable immune responses.

Natural infection with the influenza virus generates a multi-faceted immune response that can provide heterologous and/or heterosubtypic protection against influenza disease [8,9,10]. Recent recommendations emphasize that the next generation of influenza vaccines should aim to elicit similar systemic and mucosal immune responses in order to increase VE [8,11]. Currently available inactivated influenza vaccines (IIV) primarily induce neutralizing antibodies against the virus envelope protein hemagglutinin (HA) and depend on a close match between the vaccine and circulating viruses. Such vaccines are therefore relatively ineffective against newly emerging viruses or viruses that have drifted away from the vaccine strain. Mucosal antibody and T cell immune responses elicited after wild-type or experimental influenza virus infection have been associated with cross-protection [12,13,14,15] but are generally not elicited following vaccination with IIV [13,14,16,17].

The investigational influenza vaccine, M2SR (M2-deficient single replication), is intended to simulate wild-type influenza virus infection of the nasal mucosa, presenting a broader repertoire of influenza antigens, including the neuraminidase and internal proteins that are highly conserved among influenza viruses. The vaccine virus is produced in cells that constitutively provide the essential viral M2 protein on the virus surface but M2 is not expressed from the virus genome. The resulting virus mimics the infectious wild-type influenza virus when intranasally administered, but only for a single replication cycle, and does not produce infectious virus progeny. In animal models, M2SR vaccination thus mimics the immune responses generated following a naturally acquired, wild-type infection and induces broad-spectrum immunity, including neutralizing antibody responses to HA, mucosal and cell-mediated responses [18,19]. The intranasally administered M2SR live virus vaccine has shown homologous, heterologous and heterosubtypic protection against multiple influenza A subtypes in several animal model systems [18,19,20,21].

Here, we report the safety and immunogenicity of a prototype monovalent M2SR vaccine encoding the HA and neuraminidase (NA) of an A/Brisbane/10/2007-like H3N2 virus (further referred to here as “Bris2007 M2SR”) in a first-in-human clinical study. The H3N2 subtype was chosen as the initial target to demonstrate clinical proof-of-concept as the licensed vaccines provide sub-optimal H3N2 protection when there is a mismatch between the vaccine and circulating H3N2 strains or when multiple clades circulate simultaneously. M2SR was shown to be safe and immunogenic, inducing mucosal and systemic immune responses, including hemagglutination inhibition (HAI) antibody titers, against drifted H3N2 strains.

## 2. Materials and Methods

### 2.1. Study Vaccine and Placebo

The HA and NA gene segments of A/Brisbane/10/2007 (H3N2) were transferred into the M2SR backbone (A/Puerto Rico/8/1934, H1N1) as described previously [20]. The resulting Bris2007 M2SR vaccine was produced under good manufacturing practice conditions in the qualified production cell line M2VeroA (Vero cells that stably express the M2 protein). The vaccine virus was purified by anion exchange chromatography and formulated into SPG-NaCl buffer (303 mM sucrose, 5 mM glutamic acid, 136.9 mM sodium chloride, 2.67 mM potassium chloride, 1.47 mM potassium dihydride phosphate and 8.1 mM disodium phosphate, pH 7.2). Bris2007 M2SR (Lot# 15100251) was provided in single-use vials at a single concentration as a frozen solution that was stored at less than −65 °C until time of use. The placebo was sterile physiological saline (APP Pharmaceuticals, Schaumberg, Illinois) stored at an ambient temperature and provided in single-use packages. An unblinded pharmacist was responsible for preparing vaccine doses and filling delivery devices (MAD Nasal™ Intranasal Mucosal Atomization Device, Teleflex, Morrisville, North Carolina). Other members of the study team and laboratory staff were blinded to treatment assignment. Subjects in Cohorts 1 and 2 (10^6^ and 10^7^ TCID_50_ and placebo doses) received 0.2 mL divided approximately equally between two nares. Subjects in Cohort 3 (10^8^ TCID_50_ and placebo doses) received 0.3 mL total, 0.15 mL per nare.

### 2.2. Study Population, Design and Objectives

This was a single-center, phase 1, blinded (2 sentinel subjects dosed with unblinded M2SR preceded enrollment of each cohort), placebo-controlled, randomized, dose escalation study that enrolled 96 subjects (72 M2SR and 24 placebo recipients) into 1 of 3 sequential dose cohorts (Clinical Trials Registration: NCT02822105) from June–October in 2016. Written informed consent was provided by all participants. Healthy adults aged 18–49 were evaluated for eligibility criteria and pre-screened for A/Brisbane/10/2007 HAI titer. Subjects were randomized 3:1 M2SR to placebo and stratified by baseline (day 0) HAI titers within each dose level cohort: 10^6^ (low dose, cohort 1), 10^7^ (medium dose, cohort 2), or 10^8^ (high dose, cohort 3) TCID_50_.

The primary objective of the study was to assess the safety and tolerability of Bris2007 M2SR influenza vaccine delivered intranasally to healthy adult subjects. Secondary objectives included the evaluation of nasal virus shedding and percentage of subjects demonstrating seroconversion after Bris2007 M2SR vaccination.

### 2.3. Safety Evaluation

Subjects were monitored for adverse events (AE) for 28 days and for serious AE for 180 days after vaccination. The primary safety outcome measures in this study were the frequency and severity of local and systemic treatment-emergent AEs (TEAEs) and changes in routine hematology and chemistry parameters. Reactogenicity AEs were collected for 7 days after vaccination in a subject diary in a standard, systematic format using a grading scale based on functional assessment or magnitude of reaction. Nasal swab specimens collected on days 0 (baseline), 1, 2, 3 and 7 were evaluated for vaccine virus shedding by real-time quantitative reverse transcription polymerase chain reaction (RT-qPCR) and a culture-based infectivity assay.

### 2.4. Influenza Antibody Assays

A/Brisbane/10/2007 HA-specific IgA and IgG antibody titers were measured in serum by ELISA at FluGen as previously described [22] using recombinant A/Brisbane/10/2007 HA antigen (Immune Tech, New York, NY, USA).

A standard HAI assay was performed at Quest Diagnostics (San Juan Capistrano, CA, USA) or at FluGen to assess functional antibody levels [23]. Serum samples were treated with a receptor-destroying enzyme (RDE, Denka Seiken, Tokyo, Japan) overnight at 37 °C followed by heat inactivation for 1 h at 56 °C. Twofold dilutions of RDE-treated serum samples were incubated with influenza viruses (4 hemagglutination units per well) and 50 μL of a 0.5% suspension of turkey red blood cells (Innovative Research, Novi, Minnesota) for 30 min at room temperature. The HAI titer is reported as the reciprocal of the highest dilution that prevented hemagglutination. For individuals with a serum HAI titer < 10 at baseline, seroconversion was defined as an HAI titer ≥ 40 post-vaccination. For individuals with HAI ≥ 10 at baseline, seroconversion was defined as an HAI titer with a ≥4-fold increase from baseline post-vaccination. Seroprotection was defined as HAI ≥ 40 [24].

A/Brisbane/10/2007 HA-specific secretory IgA antibodies in nasal swab specimens were evaluated by ELISA at Saint Louis University [25]. Total IgA antibodies were evaluated using the Abnova™ IgA (Human) ELISA Kit (Fisher Scientific, Waltham, MA, USA) according to the manufacturer’s instructions.

### 2.5. Cellular Immune Response Assays

Antigen-specific IgG/IgA ELISpot assays were used to measure the frequency of anti-M2SR B-cells [26,27] at Cellular Technology Limited (CTL, Shaker Heights, OH, USA). Briefly, cryopreserved peripheral blood mononuclear cells (PBMC) were either unstimulated to measure antibody-secreting cells (ASC), or four-day pre-stimulated cultures with Resiquimod (R-848) and IL-2 (B-Poly-S, CTL) were used to measure memory B-cells. Plates were coated with H3N2 M2SR antigen overnight and thereafter washed; cells were plated at 1 × 10^6^ per well or 5 × 10^5^ cells depending on availability. Medium alone (no antigen coated) served as the negative control, and total IgG and IgA measurement served as a positive control.

Cryopreserved PBMCs were analyzed by ELISpot assay for the secretion of interferon gamma (IFN-γ) upon stimulation with influenza-specific peptides at Cellular Technology Limited (CTL, Shaker Heights, OH, USA). Briefly, PBMC plated at 4 × 10^5^ per well in triplicate onto IFN-γ-specific antibody-coated plates were stimulated with a class 1 peptide pool (Proimmune, Oxford, UK) at 2.5 or 5 µg/mL overnight and processed as described previously [28].

### 2.6. Statistical Methods

Statistical analyses were performed using SAS 9.4 or GraphPad Prism 7 (GraphPad, San Diego, CA, USA). Post-randomization stratification was used to analyze the effect of baseline HAI seropositivity. Two-tailed Fisher’s exact test was used to evaluate differences in proportions (e.g., proportions of subjects with a ≥4-fold rise in HAI titer) in each group. Results were statistically significant if *p* values were <0.05. R-squared regression analysis was used for correlation of immune responses against time.

## 3. Results

### 3.1. Demographics

**Figure 1:**
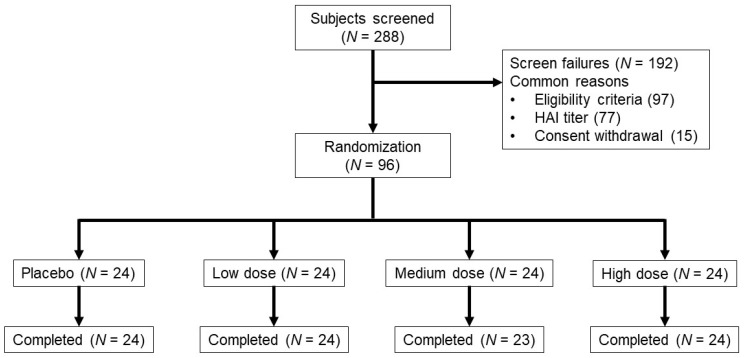
Subject disposition. Vaccine dose (TCID_50_) groups were 10^6^ (low), 10^7^ (medium) and 10^8^ (high) TCID_50_. Two subjects in each dose group were vaccinated as sentinel subjects before randomization into the group. One vaccinated subject in the medium-dose group was lost to follow up after day 2.

From June 2016 through September 2016, 288 subjects were screened. The most common reasons for screen failure (*n* = 192) were not meeting one or more of the eligibility criteria and/or high HAI titers (Figure 1). Demographic and baseline characteristics among the 96 enrolled subjects were well-balanced across the treatment groups (Table 1). One subject in the medium-dose group was lost to follow up after day 2. This subject was included in safety analyses up to day 2 but not in immunological analyses. The proportions of subjects in each baseline HAI category (<10, ≥10 to <40, and ≥40) were similar between the low and medium-dose treatment groups and placebo. More subjects (46%) had a baseline HAI ≥40 in the high-dose group compared to placebo, low and medium-dose groups (25%, 31%, 33%, respectively), but the differences were not statistically significant.

### 3.2. Vaccine Safety

All immunized participants were included in the safety analyses. All doses of Bris2007 M2SR vaccine were well-tolerated through to day 28 post-vaccination. There were no serious AEs, and no subjects withdrew due to an AE. All treatment-emergent adverse events (TEAEs) were mild or moderate in severity (Table S1). There were no subjects with severe or life-threatening TEAEs during the study. No subjects reported nasal pain or irritation after dosing during the 14-day monitoring period. No fever was observed in any subject. The most frequently reported TEAEs (all causality and treatment-related) were rhinorrhea and headache, both of which were reported at a nominally higher incidence in the medium and high-dose groups compared with placebo (although not significant for individual comparisons, Fisher’s Exact Test; *p* = 0.014 for any TEAE in the high-dose group vs. placebo) (Table S2). There were no clinically significant changes in blood and urine laboratory results during the study. Mean baseline values and mean changes from baseline in chemistry and hematology parameters were similar between the vaccinated and placebo groups.

### 3.3. Vaccine Virus Shedding

Nasal swab samples were collected from subjects at baseline (day 0, pre-dose) and on days 1, 2, 3, and 7 following intranasal vaccination. The presence of the M2SR virus was evaluated by two methods: an influenza-specific real-time quantitative RT-qPCR assay to detect viral RNA, and a culture-based infectivity assay to detect infectious viruses. Influenza virus RNA was detected in a dose-dependent manner in subjects who received the Bris2007 M2SR virus (Figure S1). Quantifiable levels of RNA (≥1900 copies/mL) were detected on day 1 in two subjects in the medium-dose group (range: 2179 to 43,919 copies/mL) and in nine subjects in the high-dose group (range: 1959 to 110,199 copies/mL), and on day 2 in one subject in the high-dose group (5491 copies/mL). RNA was not detected in any subject on day 7.

Presence of infectious virus in nasal swabs was assayed in Madin–Darby Canine Kidney (MDCK) cells that stably express M2 protein (M2CK cells), that is, cells permissive for vaccine virus growth [19]. As expected, infectious virus was not recovered at any time point tested from any of the subjects receiving Bris2007 M2SR at any dose (*n* = 71) or in the 24 placebo subjects.

### 3.4. Serum Antibody Responses

Serum antibody titers against the A/Brisbane/10/2007 virus were measured in HAI assays in all subjects before vaccination (day 0) and on days 14, 21, and 28 post-vaccination. Baseline HAI titers ranged from seronegative (<10) to seroprotected (≥40) in all cohorts (Table 1).

**Figure 2:**
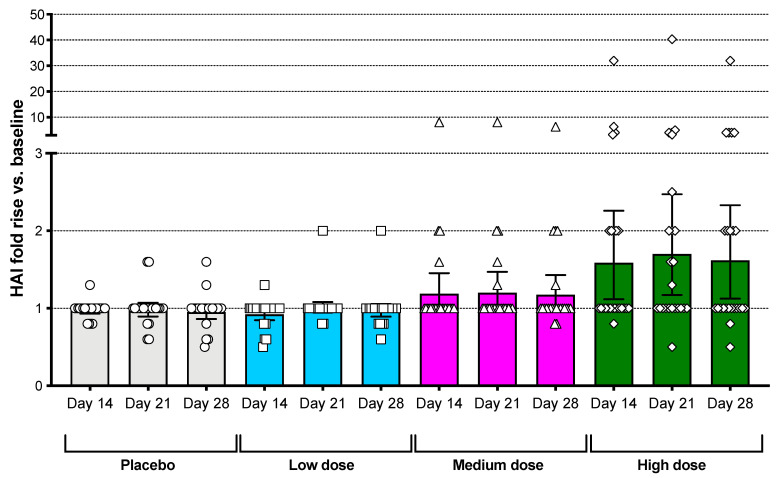
Serum HAI dose response against A/Brisbane/10/2007 virus. HAI titer fold increase vs. baseline is shown for subjects in the four different groups at days 14, 21 and 28. Bars represent the geometric mean with error bars showing the 95% confidence interval. Mean HAI titers at baseline ranged from 15 to 25.

HAI responses to A/Brisbane/10/2007 were elevated in a dose–response manner following vaccination with the Bris2007 M2SR vaccine. An increase in the number of subjects with a geometric mean fold rise (GMFR) in HAI antibody titer ≥ 2-fold compared to baseline was observed for the high-dose group only. Responses were seen as early as day 14 post-vaccination (Figure 2). In this high-dose group, HAI titers increased by at least 2-fold in 42% (10 of 24) of subjects, by at least 4-fold in 21% (5 of 24), and the protocol definition of seroconversion was met in 12.5% (3 of 24) of subjects. Most responses were seen in subjects with baseline HAI titers below 40, although two subjects with a high baseline HAI titer showed increases of at least 2-fold (Table 2), and two of the subjects with seroconversion had baseline HAI titers between 10 and 40 (Table S3). The proportion of subjects with seroprotective titers (HAI ≥ 40) at baseline in the high-dose cohort was 45.8% (95% confidence interval: 25.6–67.2%) and increased to 67% (44.7–84.4) on day 28 post-vaccination. Those subjects in the high-dose cohort with <10 HAI at baseline demonstrated 12.5% (0.3–52.7) seroprotection and those with HAI between 10 and <40 demonstrated 80.0% (28.4–99.5) seroprotection on day 28 (Table S4).

**Figure 3:**
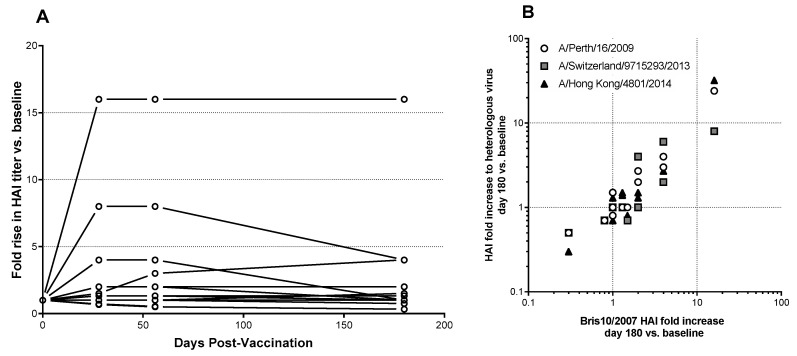
Duration and breadth of response induced by Bris2007 M2SR in high-dose cohort. (**A**) Fold increase in HAI titer against A/Brisbane/10/2007 vs. baseline at days 28, 56 and 180 post-vaccination. Lines represent individual subjects. (**B**) Correlation of fold increase in HAI titer at day 180 vs. baseline for vaccine strain A/Brisbane/10/2007 (clade 1) vs. responses to H3N2 drift strains A/Perth/16/2009 (clade 1), A/Switzerland/9715293/2013 (clade 3c3.a), and A/HongKong/4801/2014 (clade 3c2.a). R-squared values for each correlation ranged from 0.74 to 0.98 (*p* < 0.0001).

HAI titers remained elevated 6 months post-vaccination (Figure 3A) among the subset (*n* = 10) of high-dose group subjects who showed elevated HAI titers to A/Brisbane/10/2007. Furthermore, sera from these individuals were cross-reactive with more recent antigenically drifted H3N2 influenza isolates A/Perth/16/2009 (clade 1), A/Switzerland/9715293/2013 (clade 3c3.a) and A/Hong Kong/4801/2014 (clade 3c2.a) (Figure 3B). These cross-reactive antibody responses were as durable as responses against the vaccine strain (Figure 3B). The breadth of antibody response in serum was further confirmed by microneutralization assays using both the matched vaccine virus, A/Brisbane/10/2007, and A/Hong Kong/4801/2014 (data not shown, responses summarized in Table 3).

### 3.5. Mucosal Antibody Responses

**Figure 4:**
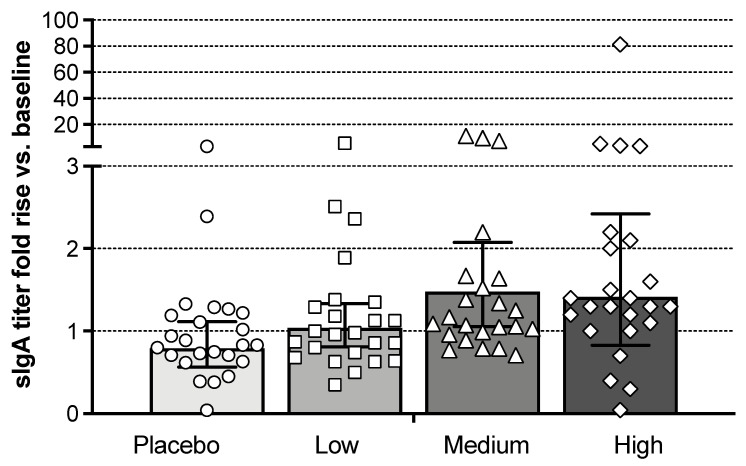
Influenza A/Brisbane/10/2007 HA-specific secretory IgA (sIgA) responses in nasal swabs. Shown are fold increase in HA-specific sIgA titer normalized to total IgA on day 28 post-vaccination over baseline. Bars represent the geometric mean ELISA titer with error bars showing the 95% confidence interval. Each symbol represents an individual subject.

Post-vaccination mucosal antibody responses on day 28 were evaluated by an influenza A/Brisbane/10/2007 HA-specific endpoint enzyme-linked immunosorbent assay (ELISA) assay for secretory IgA (sIgA) from nasal swabs. Total IgA-normalized sIgA titers relative to baseline for all cohorts on day 28 are shown in Figure 4. A dose-dependent increase in mean sIgA antibodies relative to baseline was observed. Seven of the 24 Bris2007 M2SR subjects in the high-dose cohort had a ≥2-fold rise in titers. Increases in sIgA of 2-fold or greater were observed in subjects with baseline HAI<10 (*n* = 2), seropositive (*n* = 2) and seroprotected subjects (*n* = 3). Five subjects in the high-dose group also showed an increase of more than 2-fold in serum IgA titer by ELISA compared to none in the placebo group (data not shown).

### 3.6. B Cell Responses

**Figure 5:**
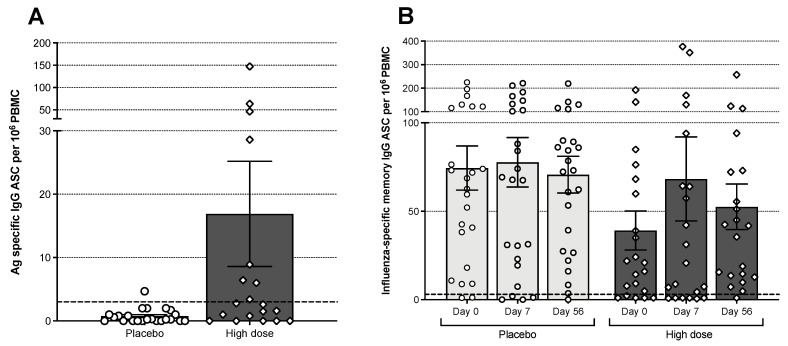
(**A**) Quantities of circulating influenza virus-specific antibody-secreting cells on day 7 post-vaccination. ASC: antibody-secreting cell. PBMC: peripheral blood mononuclear cells. Bars represent the mean with error bars showing the standard error. Number of samples analyzed: 19 for high-dose M2SR and 23 for placebo. (**B**) Influenza virus-specific memory B cells in circulation before, 7 and 56 days after vaccination. PBMC from M2SR high-dose and placebo cohorts were tested in memory B cell (IgG) ELISpot assay after four days of stimulation with B-Poly-S^TM^ for polyclonal expansion of memory B cells. Results over 3 per 10^6^ (dashed line) were considered above background.

The frequencies of influenza-specific IgG and IgA antibody-secreting (ASC) (day 7 post-vaccination) and memory B-cells (days 0, 7, and 56 post-vaccination) in PBMC from the Bris2007 M2SR high-dose and placebo cohorts were tested by direct or stimulated B-cell ELISpot assays, respectively. The M2SR high-dose cohort had a significantly higher proportion of subjects with elevated frequencies of Bris2007 Ag-specific IgG ASC B-cells at day 7 (Figure 5A) compared with placebo (63.2% vs. 16.7%, respectively, *p* = 0.0036). Interestingly, five of the subjects with elevated levels of ASC had a baseline HAI titer of 80 or higher and did not demonstrate an increase in serum antibody. Two of the high-dose M2SR subjects also demonstrated an increase in IgA ASC B-cells (data not shown). Similarly, the high-dose group demonstrated expansion of memory B-cells on days 7 and 56 post-vaccination in contrast to the placebo subjects. On day 7 and day 56, the mean fold rise in influenza specific memory cells from baseline was 1.7-fold and 1.3-fold, respectively, for the M2SR cohort compared to a 1.0-fold on day 7 and 0.95-fold on day 56 for the placebo cohort (Figure 5B).

### 3.7. T Cell Responses

**Figure 6:**
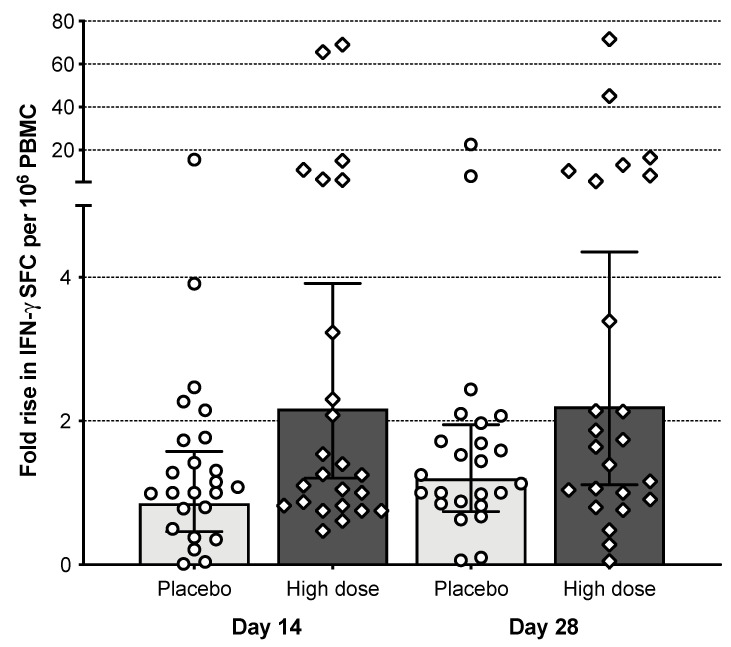
T-cell immune responses. PBMCs collected at baseline and on days 14 and 28 were analyzed by ELISpot assay for the secretion of IFN-γ upon stimulation with influenza-specific class 1 peptides. SFC: spot-forming cells. Bars represent the geometric mean with error bars.

Influenza virus-specific IFN-γ positive T-cell responses were evident on days 14 and 28 post-vaccination in subjects receiving the high dose (Figure 6). The mean fold rise in spot-forming cells (SFC) per million PBMCs from baseline to day 14 and day 28 post-vaccination was 8-fold for the M2SR high-dose cohort (both time points) and 1.8 (day 14) and 2.4 (day 28) for the placebo cohort. The number of subjects who demonstrated at least a 2-fold rise over baseline was 13 on day 14 and 12 on day 28 in the M2SR high-dose group; and 8 on day 14 and 7 on day 28 for placebo. Responses were independent of the HAI serostatus at baseline, i.e., responder subjects with pre-existing HAI titers ≥ 40 had T-cell responses similar to those in subjects with a baseline HAI < 10 (5/11 or 45%, vs. 6/8 or 75%).

### 3.8. Cumulative Immune Response of M2SR (10^8^ TCID_50_)

A summary of the various immunological assays performed for each subject in the high-dose M2SR cohort is summarized in Table 3. These results indicate that the intranasally administered M2SR stimulates multiple arms of the immune system in both seronegative and seropositive subjects. The proportion of responders (defined as a ≥2-fold increase from baseline) is shown for M2SR subjects and placebo subjects. Significant differences in responder frequencies were seen between M2SR and placebo for serum HAI, MNT and IgA responses and day 7 plasmablast ASC responses. The day 7 ASC provided the opportunity to see M2SR-elicited responses in subjects with high baseline serum HAI titers along with the mucosal sIgA responses. Overall, influenza virus-specific B cell responses were detected at a significantly higher frequency among high-dose vaccine subjects compared to placebo subjects: 87.5% (21/24) of subjects in the vaccine cohort demonstrated a ≥2-fold rise over baseline in at least one of the seven B cell assays shown in Table 3 in contrast to 45.8% (11/24) of the placebo cohort (*p* = 0.005, Fisher’s Exact Test, two-sided). Immune responses were not associated with any demographic (gender, race, ethnicity, BMI) in this small cohort.

**Table 3:** Summary of immune responses for high-dose M2SR cohort.

		SERUM				NASAL SWAB	PBMC		
Subject No.	Baseline HAI	HAI	MNT	IgG	IgA	sIgA	IgG Plasmablast	IgG ASC (Day 7, 56)	IFN-γ
1	5	**+**	-	-	-	-	-	**+**	**+**
2	5	-	**+**	-	-	-	**+**	-	-
3	5	-	-	-	-	-	-	-	-
4	5	-	-	-	-	-	-	-	**+**
5	5	-	-	-	-	-	NT	-	**+**
6	5	**+**	**+**	**+**	**+**	**+**	**+**	**+**	**+**
7	5	**+**	**+**	-	**+**	-	-	**+**	**+**
8	5	-	-	**+**	-	**+**	-	-	**+**
9	10	**+**	**+**	**+**	**+**	**+**	-	**+**	-
10	20	**+**	**+**	-	**+**	-	**+**	-	-
11	20	**+**	**+**	-	**+**	-	NT	-	**+**
12	20	**+**	**+**	-	-	-	-	**+**	-
13	20	**+**	-	-	**+**	-	NT	NT	**+**
14	40	**+**	-	-	**+**	**+**	**+**	-	-
15	40	**+**	-	-	-	-	NT	NT	**+**
16	40	-	**+**	-	-	-	NT	-	-
17	80	-	-	-	-	-	-	-	**+**
18	80	-	-	-	-	-	**+**	-	-
19	80	-	-	-	-	-	-	**+**	-
20	80	-	-	-	-	-	-	**+**	**+**
21	80	-	-	-	-	-	**+**	-	-
22	101	-	-	-	-	**+**	**+**	**+**	-
23	160	-	**+**	-	-	**+**	**+**	**+**	-
24	1280	-	-	-	-	**+**	-	-	-
**Total (% Response)**	M2SR	41.7	37.5	12.5	29.2	29.2	47.1	40.9	45.8
	Placebo	0.0	4.2	0.0	0.0	8.3	4.3	29.2	33.3
	*p* value	**0.0006**	**0.0102**	0.234	**0.0094**	0.1365	**0.0059**	0.5379	0.5556

PBMC: peripheral blood mononuclear cells; HAI: hemagglutination inhibition; MNT: microneutralization titer; ASC: antigen-secreting cells; IFN: interferon; NT: not tested. *p*-values for high dose vs. placebo cohorts, Fisher Exact Test. **+**: ≥2-fold rise from baseline; -: <2-fold rise from baseline.

## 4. Discussion

This was a first-in-human, phase 1 clinical study of a novel, live, single-replication intranasal influenza vaccine, M2SR. Single intranasal doses of M2SR, at a dose as high as 10^8^ TCID_50_, were well tolerated and elicited humoral, mucosal and cellular immune responses similar to that following natural infection [8,9,10]. Serum antibodies elicited by the M2SR vaccine were durable for at least 6 months and had broad reactivity, reacting with antigenically drifted H3N2 influenza strains from different clades and up to four years separated from the vaccine strain. Mucosal antibody, B-cell and T-cell responses were detected in seronegative and seropositive subjects, demonstrating a broad immune response independent of baseline sero-status. These results emphasize the potential for M2SR to provide improved protection against circulating strains with antigenic drift compared to currently licensed vaccines.

We assessed the antibody response to M2SR in multiple assays, including HAI and MNT, in order to further understand the immune response to this novel vaccine platform in both seronegative and seropositive subjects. We measured HAI antibody, serum IgG and IgA ELISA antibody and production of ASCs against the vaccine virus. Significant increases in serum antibodies were seen in the high-dose group, mainly in serosusceptible subjects (HAI < 40). Serum antibody increases were not detectable in subjects with high baseline HAI titers. In contrast, increases in ASC, a more sensitive measure of response to influenza vaccine [29], were seen in individuals who were seronegative and seropositive at baseline. The ASC measured on day 7 post-vaccination identified subjects who responded to M2SR vaccination but had high baseline serum HAI antibodies, as described previously for adults with high levels of baseline immunity against influenza [29]. Similarly, serum IgA may be a marker for a mucosal immune response for intranasally delivered influenza vaccines, as the baseline for IgA antibodies are lower than serum IgG levels. Past studies have found an association between serum and nasal wash IgA after intranasal administration of live influenza vaccines [16]. M2SR elicited a B cell response in 87.5% (21/24) of subjects in the high-dose cohort when all assays are considered with most subjects demonstrating a response over multiple assays. The few subjects with low or no response suggest that a dose-response plateau was not reached in this study. The safety and immunogenicity of higher doses are being evaluated in a subsequent study (NCT03999554).

The ability of M2SR to induce immune responses in seropositive individuals indicates the potential of M2SR to induce improved responses in older adults (>65 years old), a population for which the currently licensed live attenuated influenza vaccine is not indicated [30]. M2SR does not require sustained in vivo replication for generating immune responses, suggesting that the vaccine could be effective in individuals with pre-existing immunity (e.g., the elderly). The currently licensed live attenuated influenza vaccine, FluMist, is thought to be blunted by pre-existing influenza immunity present in adults, preventing sufficient vaccine virus replication to generate protective immunity and therefore less effective in adults than in children [31,32]. FluMist is not indicated in adults in Europe or in adults over 49 years old in the United States [33,34]. Pre-clinical studies in ferrets that have been pre-infected with influenza viruses have shown that M2SR is not as susceptible to pre-existing immunity as FluMist and can provide effective protection against antigenically drifted influenza strains [20]. In addition, M2SR has the desired characteristics of restimulating the existing memory pool for T cell responses, a correlate of protection in the elderly [35] that is not induced by current inactivated vaccines indicated for >65 year olds (e.g., Fluzone™ High Dose). Testing of H3N2 M2SR in older adults in a safety and immunogenicity clinical study has been initiated (clinicaltrials.gov accessed on 10 November 2021 NCT04785794).

The kinetics of HAI response in subjects showed that peak titers were reached by day 14 post-vaccination and lasted through at least 180 days post-vaccination. Moreover, these responses were cross-reactive with future H3N2 drift strains. Subjects vaccinated during the summer of 2016 with A/Brisbane/10/2007 (clade 1) had vaccine-induced HAI titers against the A/Switzerland/9715293/2013 (clade 3c.3a) vaccine strain for the 2015–2016 influenza season, and A/Hong Kong/4801/2014 (clade 3c.2), the vaccine strain for the 2016–2017 influenza season. M2SR, similar to natural infection, stimulates broadly cross-reactive antibody responses [8,9,10]. Together with local mucosal and cell-mediated immunity, M2SR has the potential to be protective against drifted H3N2, viruses unlike current inactivated vaccines that display poor VE against drift strains [3]. A subsequent Phase 2a challenge study demonstrated that M2SR provided protection against a highly drifted challenge virus [36,37].

An important attribute of M2SR is the lack of vaccine virus shedding in vaccinated subjects. Consistent with the animal data, M2SR does not produce any infectious virus after vaccination. In spite of the single-replication phenotype, vaccination with M2SR induces statistically significant levels of strain-specific antibodies in addition to mucosal and cellular immune responses. These results suggest the potential for eventual study of M2SR in young children and infants. ‘Imprinting’ of immune responses early in life have been suggested to play a critical role in future immune responses to influenza vaccines and protection against new strains [38]. Exposure to natural infection in early life was shown to provide cross-reactive immune responses to pandemic strains. However, Bodewes et al. have suggested that annual vaccination of infants with inactivated influenza vaccines prevents development of cross-reactive cellular immunity stemming from natural infection [39]. While FluMist, the only licensed live flu vaccine, does elicit T-cell responses in young children [40], it is not indicated in those less than 2 years old due to safety concerns associated with this replicating vaccine virus [30,41,42]. The single-replication M2SR induces a multi-faceted immune response against multiple influenza antigens and could potentially serve as the first immunization that ‘imprints’ for broadly reactive responses as has been suggested for natural infection [43,44,45,46,47].

## 5. Conclusions

This first human clinical trial with M2SR demonstrates that the M2-deficient influenza virus vaccine is well-tolerated and immunogenic in humans at doses up to 10^8^ TCID_50_. These phase 1 data validate the proposed target profile for M2SR, i.e., mimic wild-type infection in the nasal mucosa and induce broad humoral, mucosal and cellular responses. M2SR models the multi-faceted immune response following natural (wild-type) infection while at the same time incorporating the critical safety feature of a single round of virus replication after vaccine administration. M2SR elicited a broad immune response, including humoral, cellular, and mucosal immunity, independent of baseline HAI antibody titers. The favorable safety profile of M2SR supports continued investigation and development of this promising single-replication live vaccine, including study of higher dose levels, to address the need for broadly protective influenza vaccines in all age groups.

## Figures and Tables

**Table 1:** Baseline characteristics.

Characteristic	Placebo	Low Dose	Medium Dose	High Dose	All Treated
	(*n* = 24)	(*n* = 24)	(*n* = 24)	(*n* = 24)	(*n* = 72)
Gender (*n*, %)					
Female	13 (54%)	10 (42%)	12 (50%)	14 (58%)	36 (50%)
Male	11 (46%)	14 (58%)	12 (50%)	10 (42%)	36 (50%)
Race (*n*, %)					
Asian	0 (0%)	0 (0%)	1 (4%)	0 (0%)	1 (1%)
Black or African American	8 (33%)	7 (29%)	7 (29%)	4 (17%)	18 (25%)
White	16 (67%)	17 (71%)	16 (67%)	20 (83%)	53 (74%)
Ethnicity (*n*, %)					
Hispanic	3 (12%)	3 (12%)	4 (17%)	2 (8%)	9 (12%)
Not Hispanic	21 (88%)	21 (88%)	20 (83%)	2 (92%)	63 (88%)
Age (years)					
Mean (SD)	38.5 (7.3)	38.1 (7.1)	35.7 (6.8)	34.2 (8.2)	36.0 (7.5)
Weight (kg)					
Mean (SD)	84.1 (15.3)	84.7 (17.5)	80.4 (19.6)	84.4 (23.3)	83.2 (20.1)
BMI (kg/m^2^)					
Mean (SD)	28.2 (4.9)	28.8 (5.0)	27.4 (5.2)	29.1 (6.3)	28.4 (5.5)
Baseline HAI (*n*, %)					
<10	6 (25%)	5 (21%)	5 (21%)	8 (33%)	18 (25%)
≥10 and <40	12 (50%)	14 (58%)	11 (46%)	5 (21%)	30 (42%)
≥40	6 (25%)	5 (21%)	8 (33%)	11 (46%)	24 (33%)

SD: standard deviation of the mean; BMI: body mass index; HAI: hemagglutination inhibition.

**Table 2:** Day 28 HAI responses rates after high-dose vaccination.

	Placebo			High Dose M2SR		
Baseline HAI	Seroconversion ^a^	≥4-Fold Increase ^b^	≥2-Fold Increase ^b^	Seroconversion	≥4-Fold Increase	≥2-Fold Increase
<40	0% (0/18)	0% (0/18)	0% (0/18)	23.1% (3/13)	39% (5/13) ^c^	62% (8/13) ^c^
≥40	0% (0/6)	0% (0/6)	0% (0/6)	0% (0/11)	0% (0/11)	18% (2/11)
All	0% (0/24)	0% (0/24)	0% (0/24)	12.5% (3/24)	21% (5/24) ^b^	42% (10/24) ^d^

^a^ Percentage (number) with ≥4-fold increase from baseline and with day 28 HAI titer ≥40 post-vaccination; ^b^ Percentage (number) with day 28 HAI titer at least 4-fold or 2-fold greater than baseline; ^c^ Fisher’s exact test *p* < 0.05 vs. placebo; ^d^ Fisher’s exact test *p* < 0.01 vs. placebo.

## Supplementary Materials

The following are available online at https://www.mdpi.com/article/10.3390/vaccines9121388/s1, Supplemental Data, Table S1: Overall Summary of Adverse Events (Safety Set); Table S2: Most Frequent Treatment-Related Treatment-Emergent Adverse Events; Table S3: Seroconversion at Day 28 by Treatment Group and Baseline HAI Titer; Table S4: Seroprotection at Day 28 by Treatment Group and Baseline HAI Titer; Figure S1: Nasal shedding (RT-PCR).
