# Supplementary material for: Safety and Immunogenicity of M2-Deficient, Single Replication, Live Influenza Vaccine (M2SR) in Adults
Source: Vaccines (Basel). 2021 Nov 24;9(12):1388. doi: 10.3390/vaccines9121388 (PMC8707871; doi:10.3390/vaccines9121388)
Supplement: Supplementary file 1 [file vaccines-09-01388-s001.zip › vaccines-1450049-supplementary.pdf]

Supplemental Data

Table S1. Overall Summary of Adverse Events (Safety Set).

	Placebo (*N* = 24) *n* (%)	1x10[6] (TCID$_{50}$) (*N* = 24) *n* (%)	1x10[7] (TCID$_{50}$) (*N* = 24) *n* (%)	1x10[8] (TCID$_{50}$) (*N* = 24) *n* (%)	Total Treated (*N* = 72) *n* (%)
Subjects with at least one TEAE	16 (67)	14 (58)	21 (88)	23 (96)	58 (81)
Subjects with at least one TEAE Related to IP	11 (46)	7 (29)	14 (58)	20 (83)	41 (57)
Subjects with TEAEs by worst severity					
Mild	14 (58)	11 (46)	20 (83)	18 (75)	49 (68)
Moderate	2 (8)	3 (12)	1 (4)	5 (21)	9 (12)
Severe	0	0	0	0	0
Life-threatening	0	0	0	0	0
Subjects with TEAEs Related to IP by worst severity					
Mild	10 (42)	6 (25)	14 (58)	18 (75)	38 (53)
Moderate	1 (4)	1 (4)	0	2 (8)	3 (4)
Severe	0	0	0	0	0
Life-threatening	0	0	0	0	0

Abbreviations: IP = investigational product; TCID$_{50}$ = tissue culture infectious dose, 50%; TEAE = treatment-emergent adverse event

Table S2. Most Frequent Treatment-Related Treatment-Emergent Adverse Events Reported in at Least 5% of Subjects in the Total Treated Group by System Organ Class and Preferred Term (Safety Set).

MedDRA System Organ Class Preferred Term	Placebo (*N* = 24) *n* (%)	1x10[6] (TCID$_{50}$) (*N* = 24) *n* (%)	1x10[7] (TCID$_{50}$) (*N* = 24) *n* (%)	1x10[8] (TCID$_{50}$) (*N* = 24) *n* (%)	Total Treated (*N* = 72) *n* (%)
Total Subjects with Any Related TEAE	11 (46)	7 (29)	14 (58)	20 (83) *	41 (57)
Respiratory, thoracic and mediastinal disorders	8 (33)	5 (21)	12 (50)	19 (79)	36 (50)
Rhinorrhoea	7 (29)	1 (4)	9 (38)	10 (42)	20 (28)
Nasal congestion	3 (12)	2 (8)	2 (8)	7 (29)	11 (15)
Cough	0	2 (8)	1 (4)	2 (8)	5 (7)
Oropharyngeal pain	0	0	1 (4)	3 (12)	4 (6)
Nervous system disorders	4 (17)	3 (12)	5 (21)	5 (21)	13 (18)
Headache	4 (17)	3 (12)	5 (21)	5 (21)	13 (18)
General disorders and administration site conditions	2 (8)	2 (8)	2 (8)	3 (12)	7 (10)
Fatigue	1 (4)	2 (8)	1 (4)	1 (4)	4 (6)

Abbreviations: TCID$_{50}$ = tissue culture infectious dose, 50%; TEAE = treatment-emergent adverse event. * Total subjects in high dose group with TEAE, $p = 0.014$ FET vs placebo; individual symptom comparisons not significant.

Table S3. Seroconversion [a] at Day 28 by Treatment Group and Baseline HAI Titer.

Baseline HAI	Placebo	Low dose	Medium dose	High dose
All Subjects	0/24<br>(0.0%, 0.0–14.2)	0/24<br>(0.0%, 0.0–14.2)	1/23<br>(4.3%, 0.1–21.9)	3/24<br>(12.5%, 2.7–32.4)
<10	0/6<br>(0%, 0.0–45.9)	0/5<br>(0%, 0.0–52.2)	0/5<br>(0%, 0.0–52.2)	1/8<br>(12.5%, 0.3–52.7)
≥10 and <40	0/12<br>(0%, 0.0–26.5)	0/14<br>(0%, 0.0–23.2)	1/11<br>(9.1%, 0.2–41.3)	2/5<br>(40.0%, 5.3–85.3)
≥40	0/6<br>(0%, 0.0–45.9)	0/5<br>(0%, 0.0–52.2)	0/7<br>(0%, 0.0–41.0)	0/11<br>(0%, 0.0–28.5)

[a] seroconversion is defined as HAI titer <10 at baseline and ≥40 post-vaccination, OR HAI ≥ 10 at baseline with ≥4-fold increase from baseline post-vaccination. Shown (%, 95% confidence interval).

Table S4. Seroprotection [a] at Day 28 by Treatment Group and Baseline HAI Titer.

Baseline HAI	Placebo	Low dose	Medium dose	High dose
All Subjects	6/24<br>(25.0%, 9.8–46.7)	5/24<br>(20.8%, 7.1–42.2)	8/23<br>(34.8%, 16.4–57.3)	16/24<br>(66.7%, 44.7–84.4)
<10	0/6<br>(0%, 0.0–45.9)	0/5<br>(0%, 0.0–52.2)	0/5<br>(0%, 0.0–52.2)	1/8<br>(12.5%, 0.3–52.7)
≥10 and <40	1/12<br>(8.3%, 0.2–38.5)	0/14<br>(0%, 0.0–23.2)	1/11<br>(9.1%, 0.2–41.3)	4/5<br>(80.0%, 28.4–99.5)
≥40	5/6<br>(83.3%, 35.9–99.6)	5/5<br>(100%, 47.8–100.0)	7/7<br>(100%, 59.0–100.0)	11/11<br>(100%, 71.5–100.0)

[a] seroprotection is defined as HAI ≥ 40 at day 28.Shown (%, 95% confidence interval).

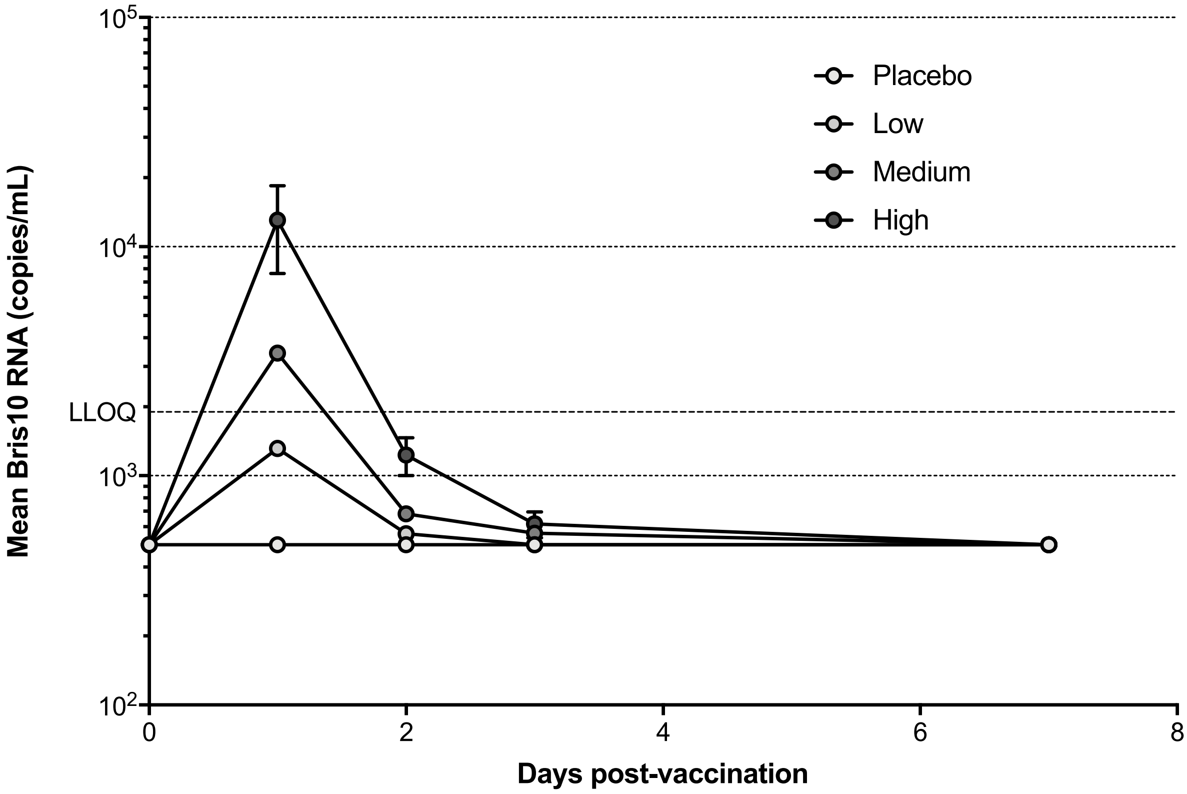

Figure S1. Nasal shedding (RT-PCR).

Cohort	N	Day 0	Day 1	Day 2	Day 3	Day 7
Placebo	24	0	0	0	0	0
Low	24	0	14 (0)[a]	2 (0)	0	0
Medium	23	0	18 (2)	3 (0)	1 (0)	0
High	24	0	23 (9)	10 (1)	2 (0)	0

[a] number of swabs with detectable (quantifiable) M2SR RNA by qPCR.

Figure S1. Mean RNA levels for each cohort at day 1, 2, 3 and 7 post-vaccination are shown. Individual results below the lower limit of quantitation (LLOQ) were assigned a value of 500.
